# Identification of gene biomarkers for brain diseases via multi-network topological semantics extraction and graph convolutional network

**DOI:** 10.1186/s12864-024-09967-9

**Published:** 2024-02-14

**Authors:** Ping Zhang, Weihan Zhang, Weicheng Sun, Jinsheng Xu, Hua Hu, Lei Wang, Leon Wong

**Affiliations:** 1 College of Information Science and Engineering, Zaozhuang University, Zaozhuang, 277100 Shandong China; 2 Guangxi Key Lab of Human-Machine Interaction and Intelligent Decision, Guangxi Academy of Sciences, Nanning, 530007 China; 3 College of Big Data and Internet, Shenzhen Technology University, Shenzhen, 518118 China; 4 CAS Key Laboratory of Plant Germplasm Enhancement and Specialty Agriculture, Wuhan Botanical Garden, The Innovative Academy of Seed Design, Chinese Academy of Sciences, Hubei Hongshan Laboratory, Wuhan, 430074 China; 5 College of Informatics, Huazhong Agricultural University, Wuhan, 430070 China

**Keywords:** Gene biomarkers, Brain diseases, Gene-disease associations prediction, Multi-network topological semantics, Graph convolutional network

## Abstract

**Background:**

Brain diseases pose a significant threat to human health, and various network-based methods have been proposed for identifying gene biomarkers associated with these diseases. However, the brain is a complex system, and extracting topological semantics from different brain networks is necessary yet challenging to identify pathogenic genes for brain diseases.

**Results:**

In this study, we present a multi-network representation learning framework called M-GBBD for the identification of gene biomarker in brain diseases. Specifically, we collected multi-omics data to construct eleven networks from different perspectives. M-GBBD extracts the spatial distributions of features from these networks and iteratively optimizes them using Kullback–Leibler divergence to fuse the networks into a common semantic space that represents the gene network for the brain. Subsequently, a graph consisting of both gene and large-scale disease proximity networks learns representations through graph convolution techniques and predicts whether a gene is associated which brain diseases while providing associated scores. Experimental results demonstrate that M-GBBD outperforms several baseline methods. Furthermore, our analysis supported by bioinformatics revealed *CAMP* as a significantly associated gene with Alzheimer's disease identified by M-GBBD.

**Conclusion:**

Collectively, M-GBBD provides valuable insights into identifying gene biomarkers for brain diseases and serves as a promising framework for brain networks representation learning.

**Supplementary Information:**

The online version contains supplementary material available at 10.1186/s12864-024-09967-9.

## Background

According to the Global Burden of Disease study, brain diseases have emerged as the leading cause of disability and the second leading cause of death since 2016 [1], imposing a substantial burden on individuals and society [2, 3]. As the intricate central nervous system organ, the brain orchestrates every bodily process. Sustaining a healthy brain is imperative for attaining longevity and overall well-being [4]. However, diagnosing and treating brain diseases pose complex challenges [5–8]. Numerous human brain diseases exhibit significant genetic components [9–11]. Identifying gene biomarkers associated with these conditions is crucial for elucidating their pathogenesis and facilitating drug development. Consequently, this can enable early clinical diagnosis and treatment.

Identification of gene biomarkers for diseases is typically achieved through linkage analysis [12, 13], large clinical cohorts [14, 15], and genome-wide association studies (GWAS) [16, 17]. However, these approaches are time-consuming and costly, particularly in the context of brain diseases. It should be noted that genes require complex regulation to perform biological functions and diseases rarely result from a single gene abnormality [18–20]. Several network-based strategies have been proposed for disease gene prediction and have successfully been applied to the study of brain diseases [21–27]. For instance, the MAGI method utilizes random walk techniques to integrate protein–protein interactions and co-expression networks during brain development to identify genes associated with autism and intellectual disability [22]. Another example is eMAGMA which incorporates genetic and expression networks into tissue-specific analyses to identify genes related to depression risk [28]. In addition to the molecular-based network studies mentioned above, several investigations have focused on brain functional connectivity (BFC) networks constructed using functional magnetic resonance imaging (fMRI). Nevertheless, it is important to note that these methods primarily focus on a single network without providing a comprehensive overview of information across multiple types of networks.

Integrating multiple types of networks allows for the combination of multi-dimensional information, compensating for the limitations of a single network [29, 30]. However, effectively leveraging diverse biological networks to identify disease-related genes remains challenging due to their spatial inconsistencies and high structural heterogeneity. Given the complexity of the brain and its requirement for precise gene biomarker prediction, a comprehensive fusion of multiple networks is necessary [31]. The BFC network reflects functional correlations between genes in the brain [32]. A framework called brainMI has been developed to enable consistent representation of BFC and molecular networks, facilitating predictions on gene-brain disease associations using machine learning approaches [7]. However, the gene network used by brainMI is solely an inference network derived from matrix multiplication. Consequently, this approach overlooks gene regulatory relationships and lacks comprehensiveness in terms of fusion. Therefore, it is crucial to fully consider transcription factor regulation when constructing a biologically meaningful gene network.

Regulatory interactions between transcription factors (TFs) and their targets constitute a gene regulatory network (GRN), which is pivotal for understanding the mechanisms underlying various biological processes [33–35]. With advancements in sequencing technologies, numerous large-scale projects have implemented bulk or single-cell RNA sequencing, resulting in an extensive collection of gene regulation data [34–36]. Hence, integrating TFs to enhance the accuracy of gene networks has become both feasible and increasingly urgent, particularly for complex brain diseases. Furthermore, from the perspective of constructing rugged networks, introducing intermediate/bridge nodes can effectively mitigate noise associated with network connections and minimize the presence of pseudo-edges within the network to some extent [37, 38]. Additionally, different diseases exhibit shared similarities that enable construction of a disease proximity network. Previous studies have demonstrated that genes associated with similar diseases are more likely to possess physical interactions among their protein products as well as display similar expression patterns [39, 40]. In conclusion, modeling the brain network as an association network comprising genes and diseases can effectively and directly reflect the correlation between brain diseases and genes implicated in causing these disorders. This approach can be regarded as a link prediction issue within complex networks. Identification of gene-level biomarkers for brain diseases will provide novel insights into causative genes identification, drug repositioning and disease taxonomy.

In recent years, deep learning methods, especially Graph Neural Networks (GNN) based methods, have been widely used in brain network studies [41–44]. It is advantageous to use GNNs due to their power to combine node features and graph structures through end-to-end feature combinations and model the adjacency relationship between nodes via message passing [45]. Among GNNs, Graph Convolutional Network (GCN) [46] stands out as a typical method that leverages structure information and performs convolution operations on graphs to aggregate neighboring node features. Given the diverse, informative, and complex nature of brain networks, it is reasonable and efficient to perform link prediction tasks by fusing multiple heterogeneous networks. Consequently, several methods have been proposed to employ GCN for learning latent patterns in brain networks for purposes such as brain disease classification or identification of related genes [47–50]. However, existing methods are limited by their usage of restricted diseases and networks within large and complex brain networks, thus hindering the potential for predicting related pathogenic genes.

In this study, we propose M-GBBD, a Multi-network representation learning framework for the identification of Gene Biomarkers in Brain Disease. We employ eleven brain networks and extract topological semantics using a joint optimizer with dual feature extraction channels to comprehensively capture brain features. By incorporating a disease proximity subgraph and gene-disease bipartite graph into a heterogeneous graph obtained by M-GBBD, we obtain a brain gene network with biological significance. The GCN is then utilized to learn representations of gene and neurodegenerative diseases from the heterogeneous graph, enabling the prediction of association scores between genes and brain diseases. Comprehensive experimental results demonstrate that M-GBBD achieves highly competitive performance compared to several baselines in terms of both dataset and model architecture. Importantly, the generalizability and accuracy of M-GBBD are confirmed by large-scale cohort GWAS studies, where we identify *CAMP* as a potential candidate gene associated with Alzheimer's disease.

## Materials and methods

### Overview of networks used in M-GBBD

**Fig. 1:**
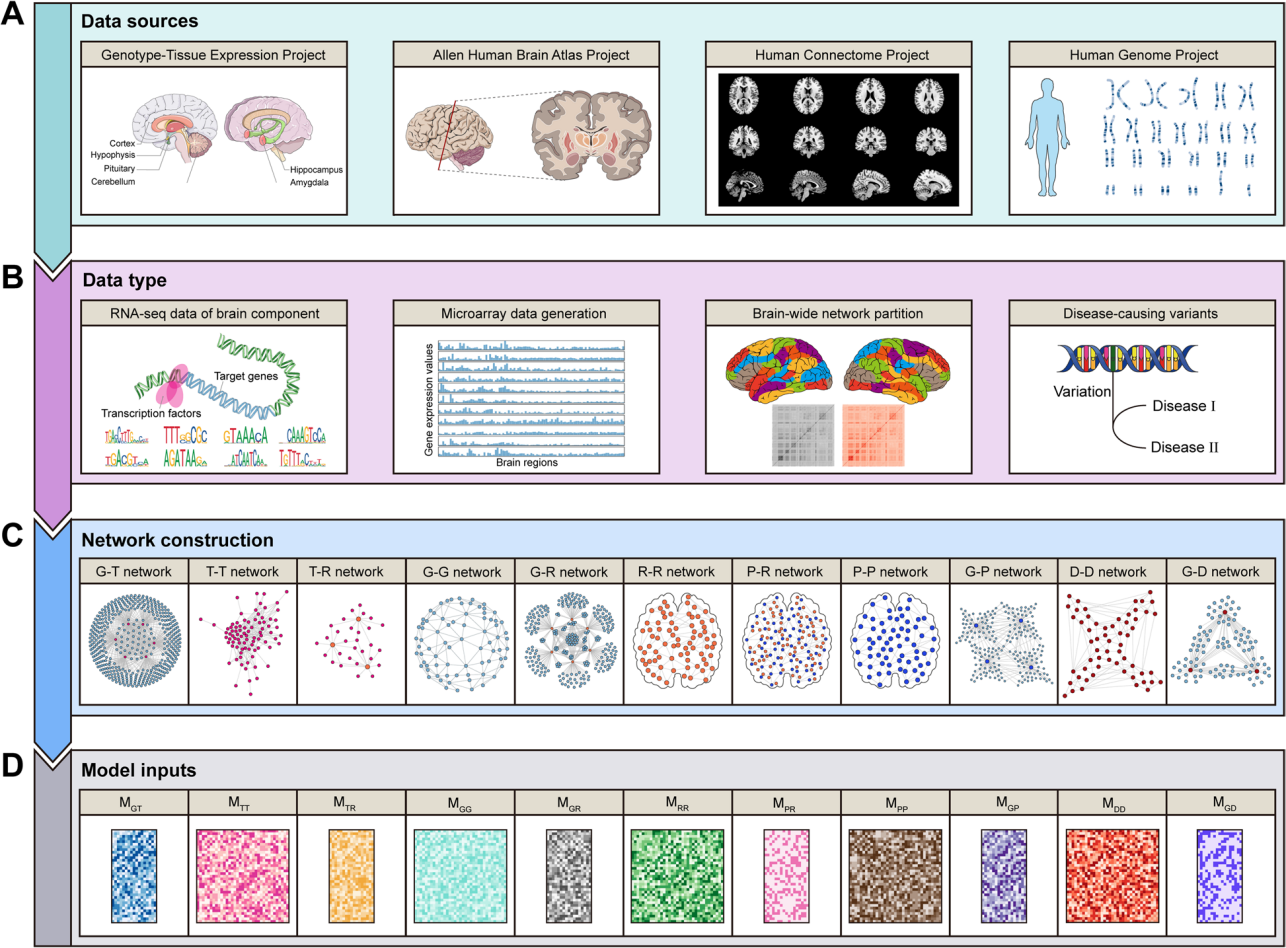
Overview of the datasets used in M-GBBD. **A** The data sources for each project. **B** The types of raw data collected for each project. **C** Various brain networks constructed using the collected data. **D** Mathematical representations in the form of unique matrices are used to represent each brain network as inputs for M-GBBD

This study employe four types of omics data, including genomics, transcriptomics, radiomics, and connectomics to construct distinct brain networks for training and testing our model. The genomic data include human genome sequence and gene annotation information as well as disease pathogenic variants, obtained from the Human Genome Resources at NCBI (version GRCh38) and DisGeNET database [51]. The transcriptomic data consist of two types of gene expression datasets downloaded from Allen Human Brain Atlas (AHBA) [52] and Genotype-Tissue Expression (GTEx) [53], along with gene regulatory data downloaded from Gene Regulatory Networks Database (GRNdb) [34]. Radiomic data comprise brain r-fMRI signals obtained from Human Connectome Project (HCP) [54]. Regarding connectomic data, we obtained the brain functional connectivity network framework developed by the Cole Neurocognition Lab [55] (Fig. 1).

A total of eleven brain networks are constructed in this study (Table 1 and Supplemental Notes): Gene regulatory network (G-T), TF-TF similarity network (T-T), TF and brain region matching network (T-R), Gene network based on regulatory relationships (G-G), Gene-region expression network (G-R), Brain region-region functional connectivity network (R-R), Brain parcel and region matching network (P-R), Brain parcel-parcel functional connectivity network (P-P), Gene-parcel expression network (G-P), Disease-disease similarity network (D-D) and Gene-disease association network (G-D).

**Table 1:** Summary of brain networks used in this study

Brain networks	Mathematical representations	Nodes	Edges
G-T	M_G-T_	14,923	123,045
T-T	M_T-T_	728	15,714
T-R	M_T-R_	4,430	2,687,652
G-G	M_G-G_	14,195	105,824,555
G-R	M_G-R_	17,897	52,549,890
R-R	M_R-R_	3,702	13,704,804
P-R	M_P-R_	4,420	2,933
P-P	M_P-P_	718	515,524
G-P	M_G-P_	14,913	5,706,390
D-D	M_D-D_	10,392	992,230
G-D	M_G-D_	24,587	588,178

### Overview of M-GBBD

**Fig. 2:**
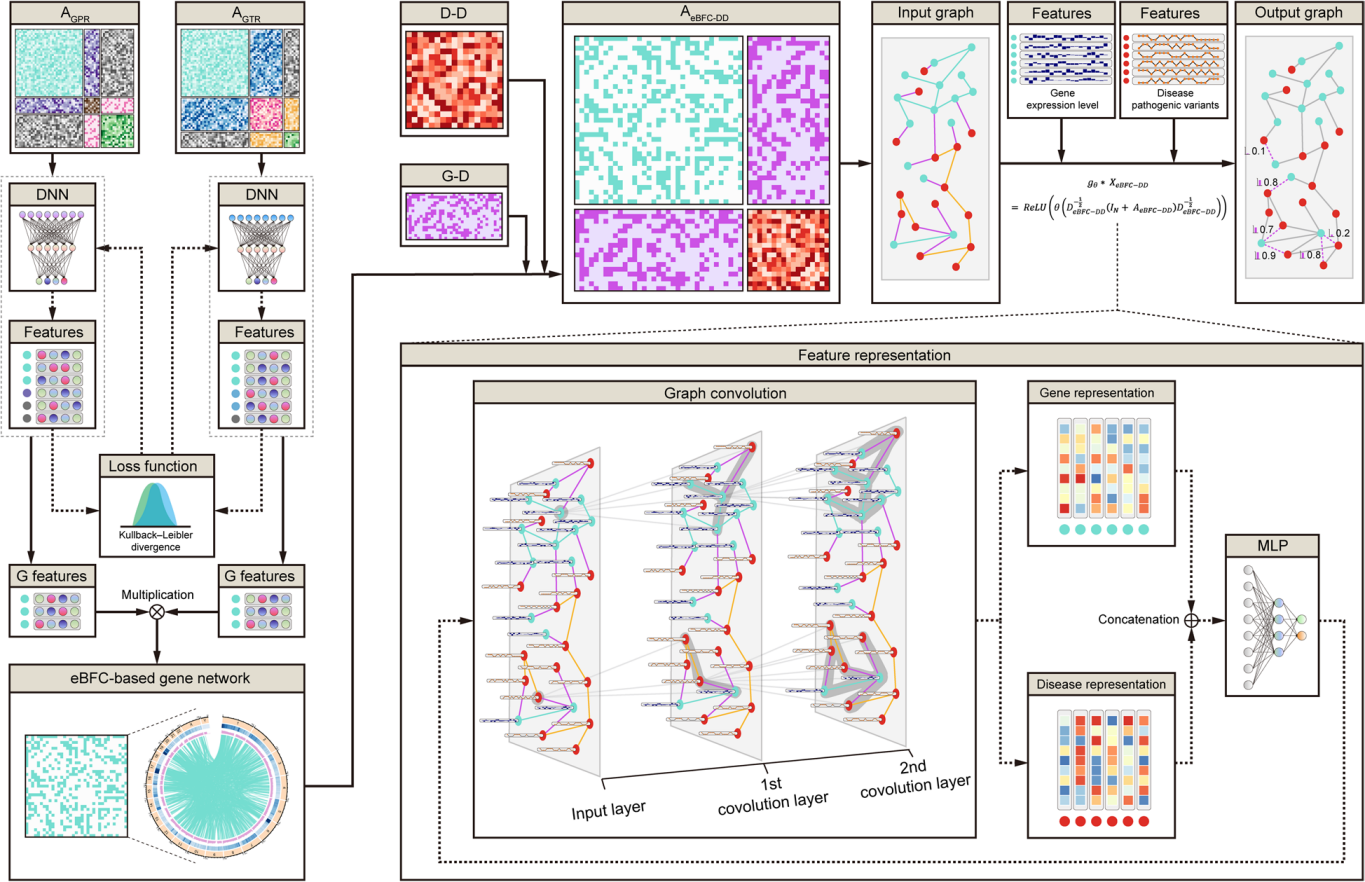
Overview of the M-GBBD framework. The framework takes two brain heterogeneous graphs, namely ${{\text{A}}}_{{\text{GPR}}}$ and ${{\text{A}}}_{{\text{GTR}}}$ (top left) as input. To reduce dimensionality and extract gene primary features along with spatial distributions, a multi-layer DNN is employed. The Kullback–Leibler divergence is utilized to calculate and learn the distributions of common subspace. After iterative optimization, an eBFC-based gene network is obtained. By combining the eBFC-based gene network with the D-D network and G-D network, GCN is applied to learn representations of genes and diseases. Finally, these representations are fed into MLP for predicting gene-disease associations

We model the identification of causative genes in brain diseases as a link prediction issue. M-GBBD is an end-to-end framework with three main components (Fig. 2): (i) constructing two types of brain heterogeneous graphs to comprehensively represent the brain functional connectivity and gene regulatory relationships, (ii) leveraging deep neural network (DNN) with the Kullback–Leibler (KL) divergence loss to learn topological semantics from the heterogeneous graphs, thereby generating an enhanced brain functional connectivity (eBFC)-based gene network with biological significance, and finally, (iii) integrating the eBFC-based gene network with the G-D and D-D networks to perform feature representation using graph convolution network(GCN).

To capture and integrate a richer set of structural information and features of the brain, we constructed two heterogeneous graphs. The first heterogeneous graph, denoted as ${{\text{H}}}_{{\text{GPR}}} \in {\mathbb{R}}^{\left({{\text{N}}}_{{\text{G}}}+{{\text{N}}}_{{\text{P}}}+{{\text{N}}}_{{\text{R}}}\right)\times \left({{\text{N}}}_{{\text{G}}}+{{\text{N}}}_{{\text{P}}}+{{\text{N}}}_{{\text{R}}}\right)}$, encompasses brain parcel-parcel functional connectivity, brain region-region functional connectivity and a gene network based on regulatory relationships. The second heterogeneous graph, referred to as ${{\text{A}}}_{{\text{GTR}}} \in {\mathbb{R}}^{\left({{\text{N}}}_{{\text{G}}}+{{\text{N}}}_{{\text{T}}}+{{\text{N}}}_{{\text{R}}}\right)\times \left({{\text{N}}}_{{\text{G}}}+{{\text{N}}}_{{\text{T}}}+{{\text{N}}}_{{\text{R}}}\right)}$, incorporates functional connectivity among brain regions, brain gene regulatory networks, and gene networks based on gene regulatory relationships. Mathematically, the two heterogeneous graphs can be represented by the following adjacency matrix:

1 $$\begin{array}{c}{{\text{A}}}_{{\text{GPR}}}=\left[\begin{array}{ccc}{{\text{M}}}_{{\text{GG}}}& {{\text{M}}}_{{\text{GP}}}& {{\text{M}}}_{{\text{GR}}}\\ {{{\text{M}}}_{{\text{GP}}}}^{{\text{T}}}& {{\text{M}}}_{{\text{PP}}}& {{\text{M}}}_{{\text{PR}}}\\ {{{\text{M}}}_{{\text{GR}}}}^{{\text{T}}}& {{{\text{M}}}_{{\text{PR}}}}^{{\text{T}}}& {{\text{M}}}_{{\text{RR}}}\end{array}\right]\end{array}$$

2 $$\begin{array}{c}{{\text{A}}}_{{\text{GTR}}}=\left[\begin{array}{ccc}{{\text{M}}}_{{\text{GG}}}& {{\text{M}}}_{{\text{GT}}}& {{\text{M}}}_{{\text{GR}}}\\ {{{\text{M}}}_{{\text{GT}}}}^{{\text{T}}}& {{\text{M}}}_{{\text{TT}}}& {{\text{M}}}_{{\text{TR}}}\\ {{{\text{M}}}_{{\text{GR}}}}^{{\text{T}}}& {{{\text{M}}}_{{\text{TR}}}}^{{\text{T}}}& {{\text{M}}}_{{\text{RR}}}\end{array}\right]\end{array}$$

where ${{{\text{M}}}_{{\text{GP}}}}^{{\text{T}}}$, ${{{\text{M}}}_{{\text{GR}}}}^{{\text{T}}}$, ${{{\text{M}}}_{{\text{PR}}}}^{{\text{T}}}$, ${{{\text{M}}}_{{\text{GT}}}}^{{\text{T}}}$ and ${{{\text{M}}}_{{\text{TR}}}}^{{\text{T}}}$ indicates the transpose of ${{\text{M}}}_{{\text{GP}}}$, ${{\text{M}}}_{{\text{GR}}}$, ${{\text{M}}}_{{\text{PR}}}$, ${{\text{M}}}_{{\text{GT}}}$ and ${{\text{M}}}_{{\text{TR}}}$, respectively.

### Graph topological semantics extraction

We employ a deep neural network (DNN) with the KL-divergence loss to extract topological semantics from two heterogeneous graphs. Specifically, we treat the feature maps of the two heterogeneous graphs ${{\text{A}}}_{{\text{GPR}}}$ and ${{\text{A}}}_{{\text{GTR}}}$ as two-dimensional representations and construct a joint optimizer with dual feature extraction channels. The input consists of these feature maps, which are then fed into a multi-layer DNN for dimensionality reduction and extraction of gene primary features along with their corresponding spatial distributions. Subsequently, we calculate the KL-divergence between the distributions of gene primary features to learn a common subspace that captures multiple heterogeneous information. During optimization, the DNN is iteratively trained using gradient backpropagation to enhance the representability of gene nodes, resulting in two final representation maps obtained through collaborative optimization of subspace and dual channels. These representation maps are utilized to derive an enhanced brain functional connectivity-based gene network (eBFC-based gene network), incorporating both brain functional connectivity and gene regulatory information. Following normalization based on previous studies [7], this eBFC-based gene network is further integrated into large-scale disease-disease networks to construct a bipartite graph named ${{\text{G}}}_{{\text{eBFC}}-{\text{DD}}}$. This step can be represented as follows:

3 $$\begin{array}{c}{z}^{(i,j)}={{\text{w}}}_{3}\alpha \left({{\text{w}}}_{2}{\alpha }\left({{\text{w}}}_{1}{x}^{(i,j)}+{{\text{b}}}_{1}\right)+{{\text{b}}}_{2}\right)+{{\text{b}}}_{3}\end{array}$$

where ${{\text{w}}}_{1}$, ${{\text{w}}}_{2}$ and ${{\text{w}}}_{3}$ represent the corresponding weight matrix, and ${{\text{b}}}_{1}$, ${{\text{b}}}_{2}$ and ${{\text{b}}}_{3}$ represent the bias vector for the three corresponding layers. ${\alpha }(\cdot )$ represents the activation function ReLU. The KL-divergence loss is defined as

4 $$\begin{array}{c}{\text{D}}_{\textrm{KL}}\left({\text{P}}_{\textrm{GPR}}||{\text{P}}_{\textrm{GTR}}\right)=\sum\limits_{\text{i}=1}^\textrm{n}{\text{P}}_{\textrm{GPR}}\left({\text{x}}_{\textrm{i}}\right)\text{log}\left(\frac{{\mathrm{P}}_{\mathrm{GPR}}\left({\mathrm{x}}_{\mathrm{i}}\right)}{{\text{P}}_{\textrm{GTR}}\left({\text{x}}_{\textrm{i}}\right)}\right)\end{array}$$

where ${{\text{P}}}_{{\text{GPR}}}$ and ${{\text{P}}}_{{\text{GTR}}}$ represent the distribution of different representations and

5 $$\begin{array}{c}{{\text{Z}}}_{{\text{G}}-{\text{PR}}}=f\left({{\text{Z}}}_{{\text{G}}}\cdot {{{\text{Z}}}_{{\text{PR}}}}^{{\text{T}}}\right)\end{array}$$

6 $$\begin{array}{c}{{\text{Z}}}_{{\text{G}}-{\text{TR}}}=f\left({{\text{Z}}}_{{\text{G}}}\cdot{{{\text{Z}}}_{{\text{TR}}}}^{{\text{T}}}\right)\end{array}$$

7 $$\begin{array}{c}{{\text{Z}}}_{{\text{G}}}={{\text{Z}}}_{{\text{G}}-{\text{PR}}}\cdot {{{\text{Z}}}_{{\text{G}}-{\text{TR}}}}^{{\text{T}}}\end{array}$$

where ${{\text{Z}}}_{{\text{G}}}$ denotes the representations of genes and ${{\text{Z}}}_{{\text{PR}}}$ denotes the representations of TFs and brain regions from ${{\text{Z}}}_{{\text{G}}-{\text{PR}}}$. ${{\text{Z}}}_{{\text{TR}}}$ denotes the representations of TFs and brain regions from ${{\text{Z}}}_{{\text{G}}-{\text{TR}}}$. ${\text{f}}(\cdot )$ denotes the dimension reduction operation.

### Graph convolutional network

In general, graph-based deep learning approaches can be categorized into two types: spatial-based and spectral-based. Spatial-based methods learn node representation by iteratively aggregating information from neighboring nodes, which may result in over-smoothing of the node representation [56]. On the other hand, spectral-based methods rely on the spectrum of the graph Laplacian of the design matrix [46]. Compared with spatial-based methods [57–61], spectral-based methods generally exhibit better performance in graph learning [62, 63]. A representative example of a spectral-based method is modified Chebyshev polynomials, which simplifies parameters and avoids large computational burdens. Given the complexity and scale of our networks, employing a multilayer GCN that is spectral-based to learn gene and disease representations from brain networks is feasible.

Specifically, the input to a GCN is the graph ${{\text{G}}}_{{\text{eBFC}}-{\text{DD}}}=(\mathcal{v},\mathcal{E})$, where $\mathcal{v}=({{\text{N}}}_{{\text{G}}}, {{\text{N}}}_{{\text{D}}})$ represents ${{\text{N}}}_{{\text{G}}}$ gene nodes and ${{\text{N}}}_{{\text{D}}}$ disease nodes, and $\mathcal{E}$ is a set of edges between nodes. The objective is to predict potential edges between gene-disease pairs that have not been previously identified in ${{\text{G}}}_{{\text{eBFC}}-{\text{DD}}}$. Denoting ${{\text{G}}}_{{\text{eBFC}}-{\text{DD}}}$ as an adjacency matrix ${{\text{A}}}_{{\text{eBFC}}-{\text{DD}}} \in {\mathbb{R}}^{\left({{\text{N}}}_{{\text{G}}}+{{\text{N}}}_{{\text{D}}}\right)\times \left({{\text{N}}}_{{\text{G}}}+{{\text{N}}}_{{\text{D}}}\right)}$, the features of both types of nodes are required. It should be noted that there are two types of nodes: gene nodes and disease nodes, which correspond to different types of features. For gene nodes, the features consist of gene expression levels at different brain sites based on RNA-seq results from 2,642 brain sites. Pathogenic variant genotypes are used as features for disease nodes, with a value of 1 indicating association with a variation and 0 otherwise. The raw data for both node types is encoded using stacked autoencoders (SAE) to ensure consistent feature dimensions. Denoting the dimensionality of SAE output as ${{\text{C}}}_{{\text{SAE}}}\in {\mathbb{R}}$, the final node feature matrix ${{\text{X}}}_{{\text{eBFC}}-{\text{DD}}} \in {\mathbb{R}}^{\left({{\text{N}}}_{{\text{G}}}+{{\text{N}}}_{{\text{D}}}\right)\times {{\text{C}}}_{{\text{SAE}}}}$ can be obtained by concatenating SAE outputs for gene and disease nodes.

The graph convolution is defined on a graph as the product of the input signal and the filter ${{\text{g}}}_{\uptheta }$ in the Fourier domain. Here, denoting the symmetric normalized Laplacian matrix of ${{\text{A}}}_{{\text{eBFC}}-{\text{DD}}}$ as ${{\text{L}}}_{{\text{eBFC}}-{\text{DD}}}= {{\text{U}}}_{{\text{eBFC}}-{\text{DD}}}{\Lambda }_{{\text{eBFC}}-{\text{DD}}}{{{\text{U}}}_{{\text{eBFC}}-{\text{DD}}}}^{{\text{t}}}$, where ${{\text{U}}}_{{\text{eBFC}}-{\text{DD}}}$ represents the eigenvector matrix and ${\Lambda }_{{\text{eBFC}}-{\text{DD}}}={\text{diag}}({\uplambda }_{1},{\uplambda }_{2}, {\uplambda }_{3},\dots ,{\uplambda }_{{{\text{N}}}_{{\text{G}}}+{{\text{N}}}_{{\text{D}}}})$ denotes the diagonal matrix of eigenvalues. The Fourier transform of ${{\text{X}}}_{{\text{eBFC}}-{\text{DD}}}$ can be represented as ${{{\text{U}}}_{{\text{eBFC}}-{\text{DD}}}}^{{\text{t}}}{{\text{X}}}_{{\text{eBFC}}-{\text{DD}}}$. However, computing the eigenvector matrix and eigenvalue diagonal matrix becomes computationally expensive with an increasing scale of the graph. To reduce computational complexity, a modified GCN based on Chebyshev polynomials ${{\text{T}}}_{{\text{K}}}\left({\text{x}}\right)= 2{{\text{xT}}}_{{\text{K}}-1}\left({\text{x}}\right)- {{\text{T}}}_{{\text{K}}-2}\left({\text{x}}\right)$ was used here for brain network feature representation. Consequently, we define and represent the filter ${{\text{g}}}_{\uptheta }$ as

8 $$\begin{array}{c}{{\text{g}}}_{\uptheta }\left({\Lambda }_{{\text{eBFC}}-{\text{DD}}}\right)= \sum_{{\text{K}}=0}^{{\text{K}}}{\uptheta }_{{\text{K}}}{{\text{T}}}_{{\text{K}}}\left({\widetilde{\Lambda }}_{{\text{eBFC}}-{\text{DD}}}\right)\end{array}$$

9 $$\begin{array}{c}{{\text{g}}}_{\uptheta }* {{\text{X}}}_{{\text{eBFC}}-{\text{DD}}}= \sum_{{\text{K}}=0}^{{\text{K}}}{\uptheta }_{{\text{K}}}{{\text{T}}}_{{\text{K}}}\left({\widetilde{{\text{L}}}}_{{\text{eBFC}}-{\text{DD}}}\right){{\text{X}}}_{{\text{eBFC}}-{\text{DD}}}\end{array}$$

where $\uptheta \in {\mathbb{R}}^{{\text{K}}}$ denotes the vector of Chebyshev coefficients, ${\widetilde{\Lambda }}_{{\text{eBFC}}-{\text{DD}}}=\frac{2{\Lambda }_{{\mathrm{eBFC}}-{\mathrm{DD}}}}{{\uplambda }_{{\text{max}}}}- {{\text{I}}}_{{\text{N}}}$, ${\widetilde{{\text{L}}}}_{{\text{eBFC}}-{\text{DD}}}=\frac{2{{\mathrm{L}}}_{{\mathrm{eBFC}}-{\mathrm{DD}}}}{{\uplambda }_{{\text{max}}}}- {{\text{I}}}_{{\text{N}}}$, ${{\text{I}}}_{{\text{N}}}$ denotes the identity matrix and K denotes the K^th^-order neighborhood.

Given that Chebyshev polynomials are recursive [64], the formulation is simplified by restricting K = 1 [46] and introducing activation functions in each layer (l > 0) to enhance the power of the model. Finally, the graph convolution method used in this study can be represented as

10 $$\begin{array}{c}{{\text{g}}}_{\uptheta }* {{\text{X}}}_{{\text{eBFC}}-{\text{DD}}}= \theta \left({{\text{D}}}_{{\text{eBFC}}-{\text{DD}}}^{-\frac{1}{2}}\left({{\text{I}}}_{{\text{N}}}+ {{\text{A}}}_{{\text{eBFC}}-{\text{DD}}}\right){{\text{D}}}_{{\text{eBFC}}-{\text{DD}}}^{-\frac{1}{2}}\right)\end{array}$$

11 $$\begin{array}{c}{{\text{g}}}_{\uptheta }* {{\text{X}}}_{{\text{eBFC}}-{\text{DD}}}= ReLU\left(\uptheta \left({{\text{D}}}_{{\text{eBFC}}-{\text{DD}}}^{-\frac{1}{2}}\left({{\text{I}}}_{{\text{N}}}+ {{\text{A}}}_{{\text{eBFC}}-{\text{DD}}}\right){{\text{D}}}_{{\text{eBFC}}-{\text{DD}}}^{-\frac{1}{2}}\right)\right)\end{array}$$

12 $$\begin{array}{c}\left[\genfrac{}{}{0pt}{}{{{\text{H}}}_{{\text{G}}}}{{{\text{H}}}_{{\text{D}}}}\right]={{\text{g}}}_{\uptheta }* {{\text{X}}}_{{\text{eBFC}}-{\text{DD}}}\end{array}$$

13 $$\begin{array}{c}{{\text{H}}}_{{\text{GD}}}= {{\text{H}}}_{{\text{G}}}\oplus {{\text{H}}}_{{\text{D}}}\end{array}$$

where ${{\text{D}}}_{{\text{eBFC}}-{\text{DD}}}$ denotes the diagonal matrix with diagonal entry ${[{{\text{D}}}_{{\text{eBFC}}-{\text{DD}}}]}_{{\text{i}},{\text{j}}}= \sum_{{\text{j}}}{[{{\text{A}}}_{{\text{eBFC}}-{\text{DD}}}]}_{{\text{i}},{\text{j}}}$, ${{\text{H}}}_{{\text{G}}}$ denotes the embedding of genes and ${{\text{H}}}_{{\text{D}}}$ represents the embedding of diseases. ⨁ denotes a concatenation operator and ${{\text{H}}}_{{\text{GD}}}$ denotes the embedding of gene-disease pair.

The prediction of the gene-disease association scores is formulated as an end-to-end binary classifier in this study. After applying the GCN to obtain embedding vectors, they are concatenated and used as the input for a multi-layer perception (MLP). The association scores are computed using the sigmoid function applied to the output of the last hidden layer:

14 $$\begin{array}{c}S =Sigmoid\left({{\text{W}}}_{{\text{out}}}\cdot {{\text{H}}}_{{\text{GD}}}+ {{\text{b}}}_{{\text{out}}}\right)\end{array}$$

where $\mathcal{S}$ denotes the scores of gene-disease associations, ${{\text{W}}}_{{\text{out}}}$ and ${{\text{b}}}_{{\text{out}}}$ denote the weight matrix and the bias vector.

The cross-entropy loss $\mathcal{L}$ is adopted to optimize model parameters as

15 $$\begin{array}{c}L= \sum_{{\text{i}},\mathrm{j }\in \mathcal{Y}\cup {\mathcal{Y}}^{-}}\left({{\text{y}}}_{{\text{ij}}}{\text{log}}{\widehat{{\text{y}}}}_{{\text{ij}}}+\left(1- {{\text{y}}}_{{\text{ij}}}\right){\text{log}}\left(1- {\widehat{{\text{y}}}}_{{\text{ij}}}\right)\right)\end{array}$$

where ${{\text{y}}}_{{\text{ij}}}$ represents the true label of the edges, which will be 1 or 0, $\mathcal{Y}$ and ${\mathcal{Y}}^{-}$ denote the sets of nodes contained in the positive edges set and negative edges set, respectively. Then, the whole model via back propagation algorithm in an end-to-end manner can be trained.

### Experimental setting

The prediction model is tuned using five-fold cross-validation (5-CV). To evaluate the accuracy of M-GBBD, the receiver operating characteristic (ROC) curve is employed. The area under the ROC curve (AUC) served as the primary evaluation metric. Additionally, considering AUC's bias towards imbalanced datasets, we also utilize the precision-recall (PR) curve. The area under the PR curve (AUPR) is selected as another primary evaluation metric. Besides, other evaluation metrics such as accuracy (ACC), recall (REC), precision (PRE) and F1-score (F1) are also calculated.

Several hyperparameters are consisted in the model, including the learning rate of optimizer ${\text{L}}\in \{0.0002, 0.0004, 0.0006, 0.0008\}$, the hidden dimensionality of embeddings ${\text{H}}\in \{16, 32, 64, 128\}$, the dropout rate ${\text{D}}\in \{0.01, 0.05, 0.1, 0.3\}$, the Chebyshev filter size ${\text{K}}\in \{2, 3, 4, 5\}$, and the total training epochs ${\text{E}}\in \{200, 600, 1000, 2000\}$. The best obtained parameters are ${\text{L}}=0.0004,\text{ H}=64,\text{ D}=0.05,\text{ K}=4$ and ${\text{E}}=1000$.

After intersecting all datasets used in this study, a total of 14,195 genes were retained. As we have collected comprehensive human genome-wide gene information that includes consistent characterization and network structure information here, 20 known gene-disease associations related to two specific brain diseases (Alzheimer's disease and Parkinson's disease) have been pre-isolated by random selection for further demonstration. These pre-isolated associations are not involved in any training process to prevent data leakage, and thus ensuring objectivity. Finally, a total of 14,175 genes and 10,392 diseases formed a dataset consisting of 557,893 associations which participated in the subsequent training process.

## Results

### Overall performance

**Fig. 3:**
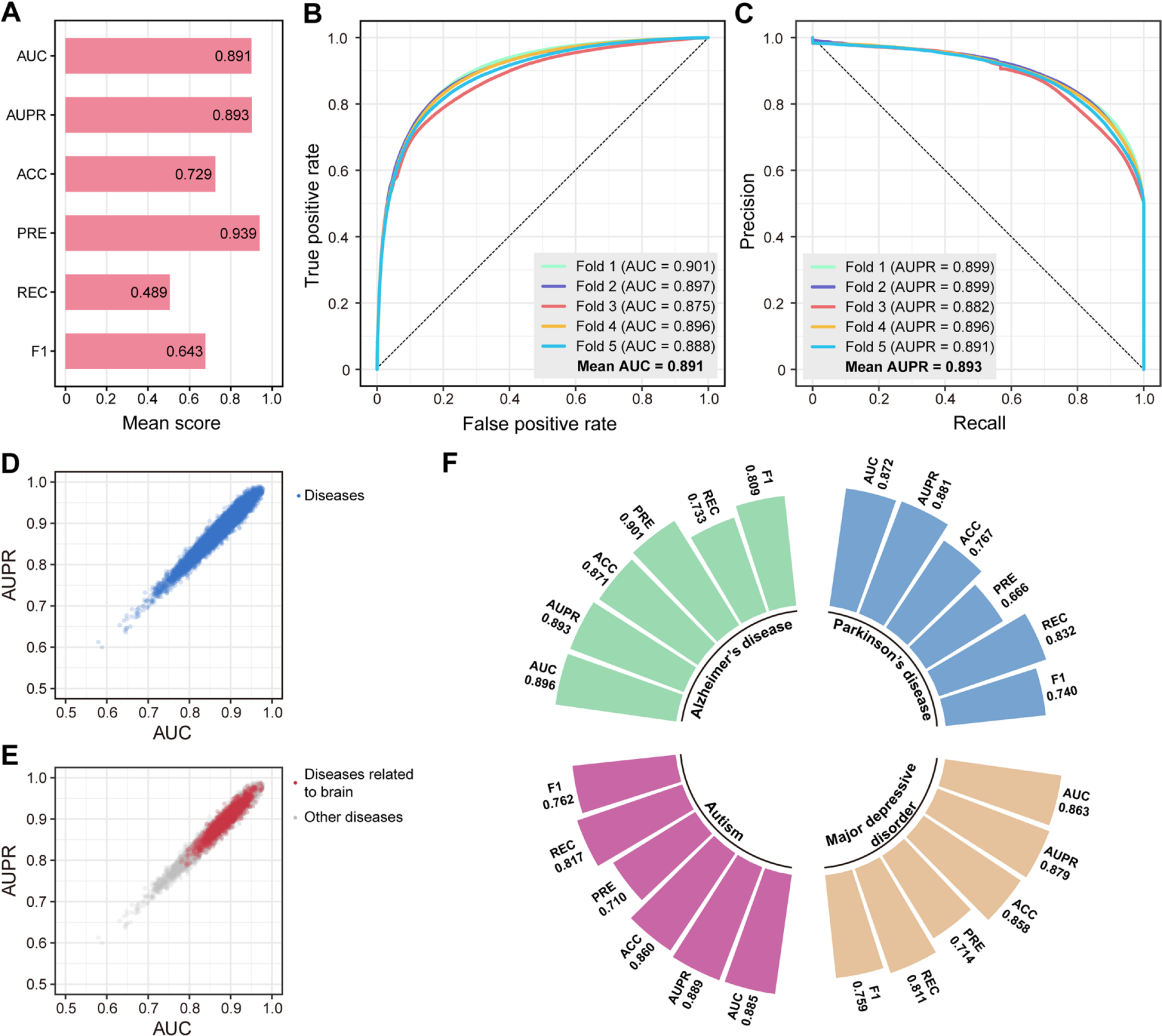
Overall performance of M-GBBD. **A** Mean values of each evaluation metrics under 5-CV. **B** ROC curves of M-GBBD under 5-CV. **C** PR curves of M-GBBD under 5-CV. **D** Distribution of AUC and AUPR values for all diseases. **E** Distribution of AUC and AUPR values for disease related to brain. **F** Performance of M-GBBD on four representative diseases that related to brain

The eBFC-based gene network, which covers most genes in the human genome, has been derived through topological semantics extraction from ${{\text{A}}}_{{\text{GPR}}}$ and ${{\text{A}}}_{{\text{GTR}}}$. It is essential to note that gene expression may be regulated through various mechanisms, resulting in one gene being associated with multiple diseases due to distinct regulatory pathways [65–67]. In other words, several common pathogenic genes can be identified across different diseases, with differential regulation of these genes being particularly prevalent among brain diseases [18, 20]. Therefore, it is more reasonable to use a link prediction paradigm for identifying pathogenic genes related to brain diseases. In our study, we constructed a disease-disease (D-D) network comprising 10,392 diseases in M-GBBD, enabling the prediction of associations between any given gene and disease within this network. Evaluation of M-GBBD performance shows that across all diseases considered, the mean values for AUC, AUPR, ACC, PRE, REC, and F1 of M-GBBD are found to be 0.891, 0.893, 0.729, 0.939, 0.489 and 0.643, respectively (Fig. 3A). Furthermore, the consistency observed in each cross-validation further supports the robustness of our finding (Fig. 3B and C). Among these 10,392 diseases, there are 2,102 kinds of diseases that are specifically associated with brain-related diseases. The AUC and AUPR values for each disease exhibit relatively similar trends (Fig. 3D). Notably, diseases linked to the brain demonstrate higher values for both AUC and AUPR compared to other non-brain related ailments (Fig. 3E), indicating that M-GBBD is sensitive to such diseases.

Furthermore, we have chosen four representative brain diseases (Alzheimer's disease, Parkinson's disease, Major depression, and Autism) for comprehensive investigation and discussion. These diseases are well-known for their high prevalence and significant impact on individuals, thus extensively studied by various models [5, 7, 8, 44, 68]. The performance evaluation metrics including AUC/AUPR/ACC/PRE/REC/F1 for the aforementioned diseases are as follows: 0.893/0.867/0.829/0.768/0.820/0.793 (Alzheimer’s disease), 0.866/0.881/0.767/0.666/0.832/0.740 (Parkinson’s disease), 0.883/0.864/0.797/0.699/0.813/0.752 (Major depressive disorder) and 0.887/0.844/0.746/0.632/0.846/0.723 (Autism), respectively (Fig. 3F). Notably, all these evaluation metrics surpass those of the previous method [7], demonstrating the excellent performance of M-GBBD.

### Improved performance of multiscale disease network and eBFC-based gene network

**Fig. 4:**
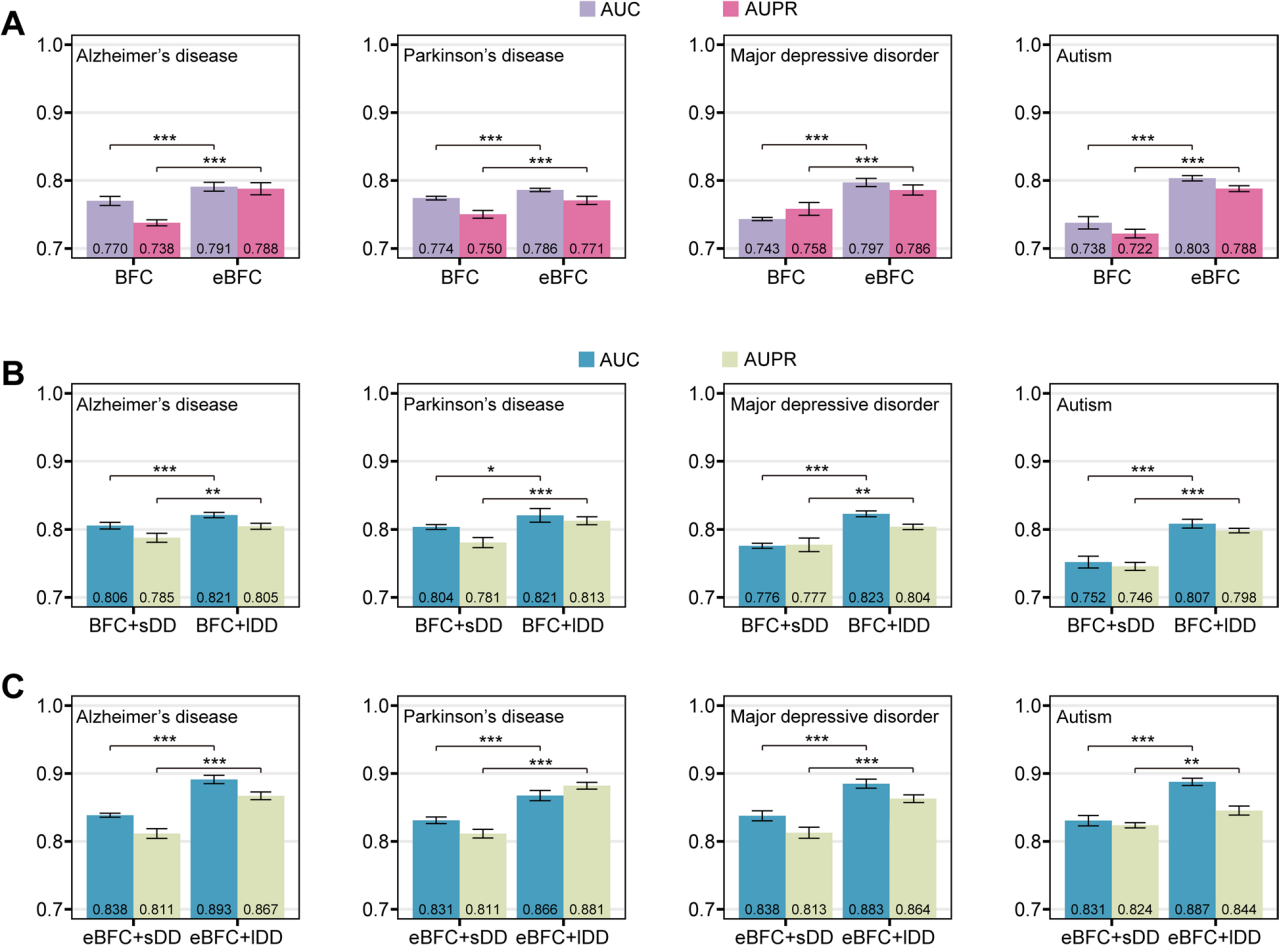
Performance of M-GBBD and brainMI with different datasets on four representative brain related diseases. **A** Mean AUC and AUPR values of brainMI with only BFC- and eBFC-based gene networks under 5-CV. **B** Mean AUC and AUPR values of M-GBBD with BFC-based gene network and sDD/lDD under 5-CV. **C** Mean AUC and AUPR values of M-GBBD with eBFC-based gene network and sDD/lDD under 5-CV. Statistical significant was estimated using two-tailed Student’s t-test. *, *P* < 0.05; **, *P* < 0.01; ***, *P* < 0.001

To evaluate the performance across different combinations of multiscale disease network and eBFC-based gene networks, we conducted three comparative experiments. The first experiment aims to evaluate the predictive performance improvement of eBFC-based gene network compared to BFC-based gene network. To be specific, we use BFC-based and eBFC-based gene networks to train and predict associations between genes and four representative brain diseases using brainMI. The results demonstrate a significantly higher performance of brainMI when utilizing eBFC-based gene network compared to BFC-based gene network (Fig. 4A). On average, the AUC and AUPR values for disease prediction by brainMI using eBFC-based gene network increase by 0.038 and 0.041, respectively, in comparison with those obtained from BFC-based gene network (Fig. 4A). This indicates that eBFC-based gene network may encompass more comprehensive information than the BFC-based counterpart, thereby improving predictive performance.

The other two experiments are conducted to evaluate the performance improvement achieved by multiscale disease networks. Due to that brainMI employs a node classification strategy whereas M-GBBD utilizes a link prediction strategy, the D-D network cannot be directly utilized in brainMI experiments. Therefore, we constructed a small-scale disease proximity network (sDD) that includes only four diseases mentioned in brainMI using the same methodology as for the D-D network and performed experiments using M-GBBD. For clarity, we refer to the D-D network used in M-GBBD as the large-scale D-D network (lDD). By combining both sDD and lDD with two gene networks (BFC-based and eBFC-based), we aim to demonstrate whether lDD can indeed improve predictive performance significantly. Compared to sDD, when combined with BFC-based gene network, lDD exhibited an average increase of 0.034 in AUC and 0.032 in AUPR, respectively (Fig. 4B). When combined with the eBFC-based gene network, there is an average improvement of 0.048 in AUC and 0.049 in AUPR using lDD (Fig. 4C). These results consistently indicate that regardless of which gene network is employed, lDD consistently outperforms sDD. In summary, utilizing the biologically significant eBFC-based gene network along with a large-scale proximity network can achieve superior performance for predicting gene-disease associations within the brain compared to traditional single BFC-based gene network.

### Comparison with the state-of-the-art frameworks

Given the tedious and multilayered nature of brain disease diagnosis, graph-based methods offer an efficient approach to learn representations for identifying associations from vast amounts of data [69, 70]. To evaluate the performance improvement of the M-GBBD algorithm, three gene-disease prediction frameworks, including BiRW [71], PMFMDA [72] and MeSHHeading2vec [73], are compared with M-GBBD. These frameworks are all designed to predict associations between genes and diseases. BiRW utilizes a bi-random walk algorithm, while PMFMDA is based on matrix factorization, and MeSHHeading2vec employs graph embedding algorithms for relationship prediction tasks. Each framework was executed using default parameters and 5-CV. The evaluation metrics including AUC, AUPR, ACC, REC, PRE, and F1 were calculated for each framework in order to facilitate comparison.

**Fig. 5:**
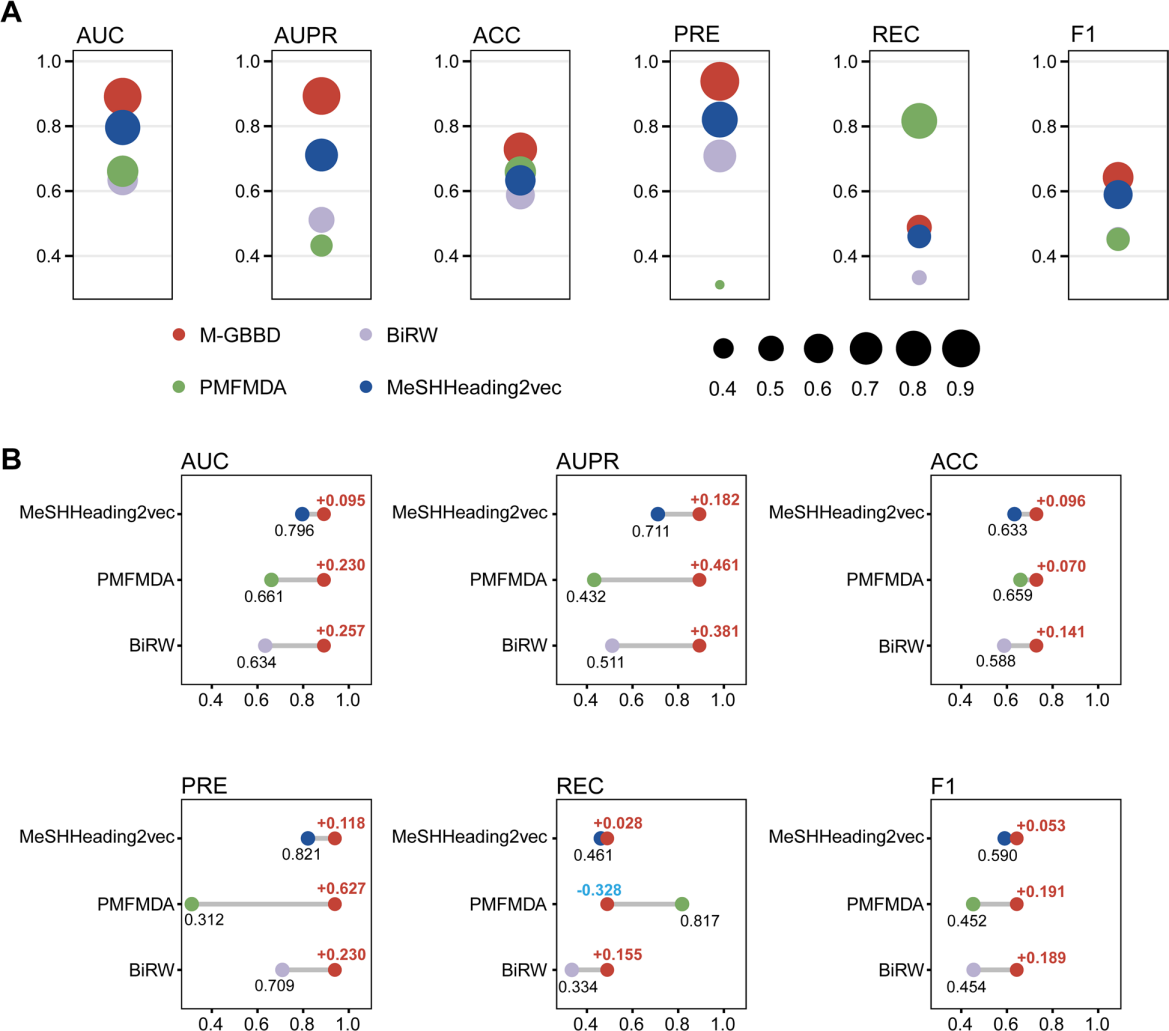
Comparison on the performance of different gene-disease prediction frameworks. **A** Results of the six evaluation metrics for the four frameworks. **B** The difference in performance of M-GBBD relative to the other three frameworks. Colors of dots are same as in (**A**) and improvement/decline are indicated by red/blue bold numbers

The results show that M-GBBD outperforms all other frameworks in terms of evaluation metrics, except for REC (Fig. 5A). Although PMFMDA achieves the highest REC value, its PRE values are the lowest. Compared to other methods, M-GBBD shows an average improvement of 0.194 and 0.341 in AUC and AUPR respectively (Fig. 5B). This superior performance can be attributed to the GCN's ability to more effectively aggregate network information. Overall, with the benefit of the GCN and its end-to-end computational structure, our M-GBBD is a more suitable method for predicting associations between genes and disease in the brain.

### Ablation analysis demonstrates the importance of multiple semantics extraction

**Fig. 6:**
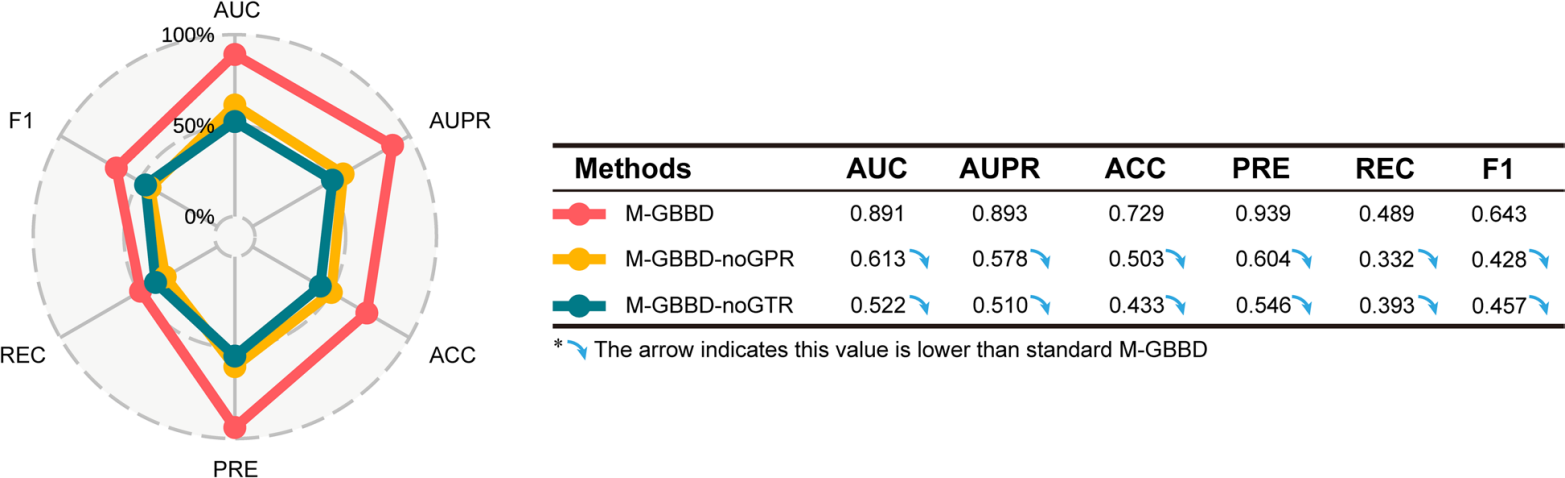
Comparison on the performance of variant methods of M-GBBD. Five evaluation metrics for the three methods including the raw M-GBBD were calculated and compared. All metrics in the table are lower than M-GBBD, which is highlighted with blue arrows

To further investigate the contribution of critical components and evaluate the robustness of M-GBBD, we compared it with two variant methods, namely M-GBBD-noGPR and M-GBBD-noGTR. The M-GBBD-noGPR method exclude the heterogeneous network comprising brain parcel-parcel functional connectivity, while the M-GBBD-noGTR method removed the heterogeneous network involving gene regulatory interactions. Following a 5-CV for each method, we obtain AUC values 0.891, 0.613 and 0.522 for M-GBBD, M-GBBD-noGPR and M-GBBD-noGTR respectively. Correspondingly, the AUPR values were found to be 0.893, 0.578 and 0.510 (Fig. 6). In addition, ACC, PRE, REC and F1 of M-GDAB are also superior to corresponding metrics of other methods (Fig. 6). Our ablation experiments results demonstrate that combining brain parcel-parcel functional connectivity with gene regulatory features forms a crucial foundation for performance improvement.

### Case studies

To demonstrate the applicability of M-GBBD in predicting potential gene-disease associations in practical scenarios, we apply M-GBBD to predict genes associated with two brain diseases: Alzheimer's disease and Parkinson's disease. For each disease, five associated genes are randomly selected while their known twenty gene-disease associations for the two diseases are concealed to ensure these associations are pre-isolated. These associations are not considered during the semantics extracting and model training steps, which make the case study objective and reliable. Subsequently, M-GBBD was used to predict the gene-disease associations for these associated genes and report their association scores. The results are validated using the DisGeNET database, based on biological experiment reports, or further bioinformatics analysis of biological data.

**Fig. 7:**
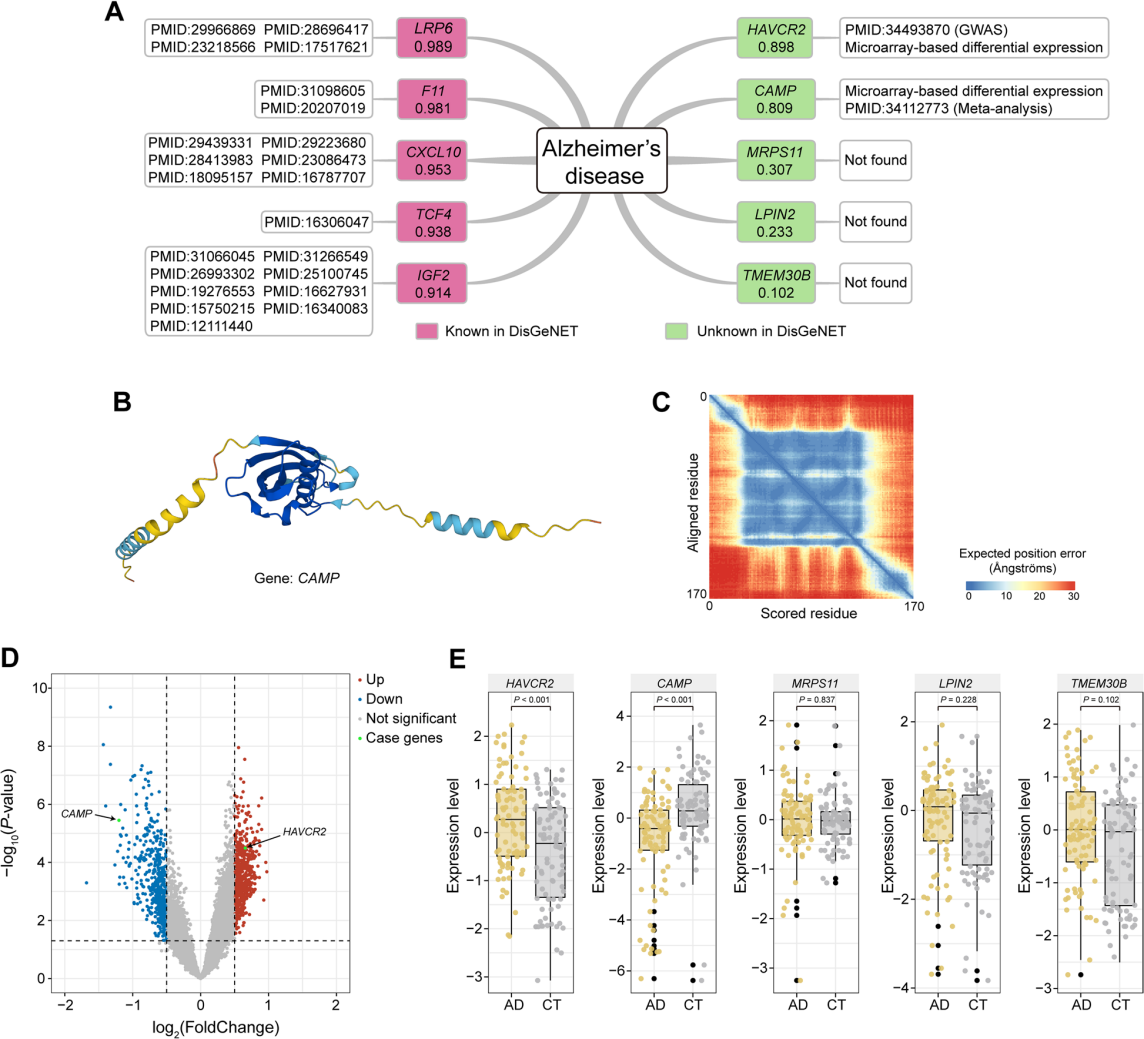
Case study of Alzheimer’s disease. **A** Gene-Alzheimer's disease associations predicted by M-GBBD, with corresponding scores. The pink box indicates that DisGeNET has recorded that this gene is associated to Alzheimer's disease, and the green box indicates that DisGeNET has no record that this gene is associated to Alzheimer's disease. The white boxes following the pink/green boxes is the evidence. **B** Three-dimensional structure of *CAMP* from AlphaFold. **C** Heatmap of the three-dimensional structure predicted aligned error. It means the AlphaFold’s expected position error reside x, which the predicted and true structures are aligned on residue y. **D** The results of differential expression analysis from microarray (GSE85426). **E** The normalized expression values of each sample in microarray (GSE85426) for five genes that not recorded in DisGeNET. Statistically significant was estimated using two-tailed Student’s t-test

In the DisGeNET database, *LRP6*, *F11*, *CXCL10*, *TCF4* and *IGF2* are identified as the top five genes associated with Alzheimer's disease, with association scores of 0.989, 0.981, 0.953, 0.938 and 0.914, respectively (Fig. 7A). Notably, all scores exceed the threshold of 0.9. Besides, *HAVCR2*, *CAMP*, *MRPS11*, *LPIN2* and *TMEM30B* are five genes without labeled associations in DisGeNET but exhibit association scores of 0.898, 0.809, 0.307, 0.233 and 0.102, respectively (Fig. 5A). Interestingly, *HAVCR2* and *CAMP* demonstrate higher scores compared to other genes, suggesting that M-GBBD has potential for predicting potential Alzheimer's disease-associated genes not yet annotated by DisGeNET. Further analysis is conducted to investigate the rationale behind the high scores of the two genes predicted by M-GBBD. According to a recent large-scale genome-wide association analysis for Alzheimer’s disease based on more than one million individuals, significant associations between *HAVCR2* and Alzheimer’s disease were found [68]. The variant site of locus 8 (rs6891966) in an intron of *HAVCR2* results in a significant differential expression level in brain tissue samples from patients compared to controls. This is consistent with the results obtained from M-GBBD, indicating an association between *HAVCR2* and Alzheimer’s disease. The protein product of *CAMP* is a sequence with 170 amino acids and the high confidence structure model was predicted by AlphaFold (Fig. 7B and C) [74]. It exhibits antibacterial activity and binds to bacterial lipopolysaccharides (LPS) [75, 76]. Although direct experimental evidence supporting the association between *CAMP* and Alzheimer's disease is currently lacking, microarray analysis (GSE85426), which included 90 patients with Alzheimer's disease and 90 controls, revealed significant changes in *CAMP* expression levels (Fig. 7D and E). Furthermore, an epigenome-wide association study also found a CpG island located in a significant differentially methylated region of *CAMP* [77]. Therefore, it is reasonable for M-GBBD to identify *CAMP* as highly associated with Alzheimer’s disease. Additionally, the microarray analysis also demonstrated significant differences in *HAVCR2* expression (*P* < 0.001) (Fig. 7E), consistent with the original report [68]. Conversely, no significant differences were observed in the expression levels of *MRPS11*, *LPIN2* and *TMEM30B* and the three genes all received low scores (Fig. 7E). Both GWAS and microarray analysis results corroborate the accuracy and applicability of M-GBBD for predicting candidate gene biomarkers related to Alzheimer's disease.

**Fig. 8:**
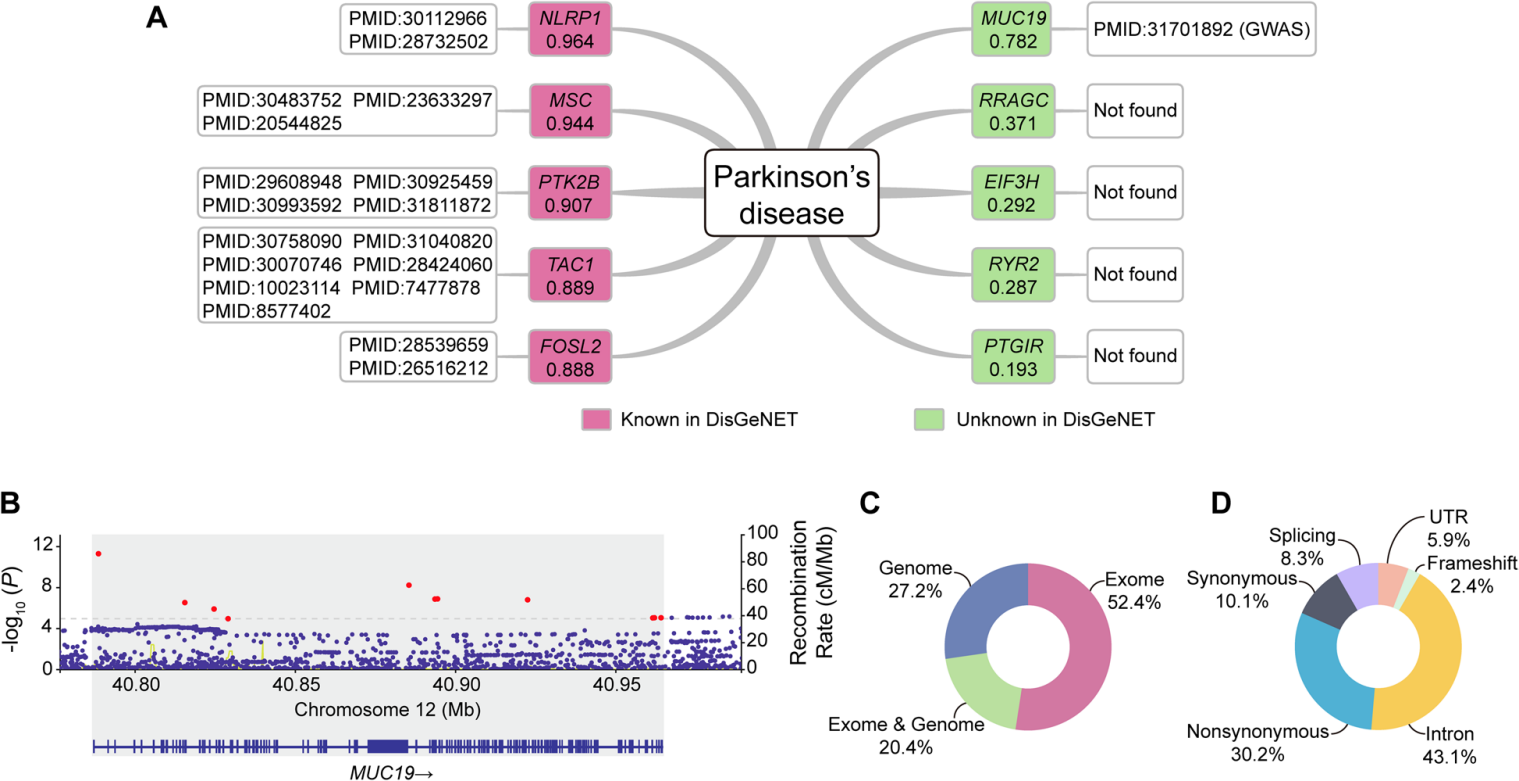
Case study of Parkinson’s disease. **A** Gene-Parkinson's disease associations predicted by M-GBBD, with corresponding scores. The pink box indicates that DisGeNET has recorded that this gene is associated to Parkinson's disease, and the green box indicates that DisGeNET has no record that this gene is associated to Parkinson's disease. The white boxes following the pink/green boxes is the evidence. **B** Manhattan plot of *MUC19*, the grey area indicates the range of the *MUC19*. Red dots are significant variants within *MUC19*. **C** The potential variant sites located or near coding region obtain from gnomAD browser. **D** The functional annotations of potential variants

In the case of Parkinson's disease, another severe neurodegenerative disorder, M-GBBD also demonstrated satisfactory performance. The DisGeNET database labels *NLRP1*, *MSC*, *PTK2B*, *TAC1* and *FOSL2* as genes associated with Parkinson’s disease, with association scores of 0.964, 0.944, 0.907, 0.889 and 0.888, respectively (Fig. 8A). Except for *MUC19* which scored at 0.782,

all other unlabeled genes have association scores below 0.4 in M-GBBD. To further investigate the potential association between *MUC19* and Parkinson’s disease, a GWAS summary based on data from 482,730 individuals and analyzing a total of 17,510,617 SNPs was collected [78]. The GWAS result revealed that there were significant associations between Parkinson's disease and eleven SNPs located within the gene body of *MUC19* (Fig. 8B), providing evidence for the relationship between *MUC19* and Parkinson’s disease. According to detailed information of *MUC19* from the human genome, 5,125 potential variant sites are located in or neighbored by gene coding region. These variants were detected by genome sequencing (27.2%), exome sequencing (52.4%) or both (20.4%) in a previous study (Fig. 8C), and 40.9% of them will cause loss of function (nonsynonymous, splicing and frameshift) (Fig. 8D) that *MUC19* was assessed to have high association with Parkinson’s disease by M-GBBD is sensible, as supported by GWAS results.

## Discussion

The brain system is a complex network of regulatory molecules, in which their interactions contribute to the normal or disordered biological characteristics of the brain system. As attention towards brain diseases increases, various graph deep learning-based studies have been proposed for brain gene biomarker identification. However, these studies have several shortcomings including limited diversity in biological network types, lack of an effective and biologically meaningful network fusion strategy, inadequate extraction of graph structure and node feature information, as well as unsatisfactory model performance and generalizability [7, 79–81]. Although we have partially addressed these limitations by developing a pioneering topological semantics extraction approach called M-GBBD to construct a biological meaningful brain gene network, this approach only extracts semantics from networks constructed using genomics, transcriptomics, radiomics, and connectomics data. Networks constructed using other omics data such as epigenomics, metabolomics and proteomics have not yet been used or discussed in this study. With advancements in molecular biology and biotechnology innovation, more comprehensive data will be easily obtained in the future. Admittedly, incorporating different types of brain networks into M-GBBD may further improve its predictive performance for associations between genes and brain diseases; however effective and accurate strategies for topological semantics extraction from brain networks that aim to obtain a gene network with rich semantics reflecting multiple biological meanings continue to pose challenges.

In addition, M-GBBD is a GCN model that follows the Transductive Learning paradigm [82], which takes a broad and global perspective on gene biomarker identification.

At the beginning of model training, the training set (nodes with edges and labels) and the node information of the test set (without edges) are available while the corresponding edge information remains unseen as these edges will be predicted in the subsequent model test phase. Although the true edges of the test set are unknown during training, additional information can be obtained from their node feature distribution, such as distribution aggregation, which resembles drug repositioning. While transductive learning can extract some additional information from all nodes and edge information in the training set to enhance model effectiveness, it also necessitates retraining and increased computation whenever new samples are received. In future work, we will further explore how to leverage inductive learning to improve identification accuracy of brain disease gene markers by considering brain network specificity.

## Conclusions

In this study, we constructed and conducted topological semantics extraction of eleven brain networks to characterize the brain features from different perspectives. In contrast to existing methods that only focus on a single disease, we introduced a biologically meaningful disease network by incorporating common disease-causing variants. Our M-GBBD model captures both functional connectivity and gene regulation information through joint optimization and multi-channel feature extraction strategies, enabling us to obtain an informative brain gene network with superior performance compared to other methods. The extraction of different network topological semantics highlights the crucial role of utilizing multi-networks for studying brain diseases comprehensively. Extensive experiments demonstrated the accuracy of M-GBBD, while case studies showcased its excellent generalizability in accurately assessing the association between genes and brain diseases. The M-GBBD gave accurate and reasonable scores for all genes used in the case analysis. Notably, our analysis suggests a potential association between *CAMP* and Alzheimer’s disease, which is further supported by in-depth bioinformatics analysis.

### Supplementary Information

**Additional file 1.**

## Data Availability

The human genome was obtained from National Center for Biotechnology Information (https://www.ncbi.nlm.nih.gov/genome/guide/human/), the diseases information and causing variants were obtained from DisGeNET (https://www.disgenet.org/home/), the gene expression data of different brain regions and sites were obtained from Allen Human Brain Atlas (https://human.brain-map.org/static/download) and Genotype-Tissue Expression project (https://gtexportal.org/home/datasets), the gene regulatory information was obtained from Gene Regulatory Networks Database (http://www.grndb.com/download/), the r-fMRI data was obtained from the Human Connectome Project (https://db.humanconnectome.org/), the CAB-NP brain functional connectivity network was obtained from the ColeLab (https://github.com/ColeLab/ColeAnticevicNetPartition). The code and data of M-GBBD are available at https://github.com/Weihankk/M-GBBD.
