# Supplementary material for: Identification of gene biomarkers for brain diseases via multi-network topological semantics extraction and graph convolutional network
Source: BMC Genomics. 2024 Feb 14;25:175. doi: 10.1186/s12864-024-09967-9 (PMC10865627; doi:10.1186/s12864-024-09967-9)
Supplement: Supplementary file 1 — Additional file 1. [file 12864_2024_9967_MOESM1_ESM.docx]

**Supplemental Notes**

**Human reference genome and annotations**

The human genome from NCBI has excellent completion and continuity with the length ~3.1Gb and contig N50 is ~57.9Mb. A total of 22,831 coding-genes information and sequences were used in this study. For more comprehensive analysis, approximately 100,000 transcripts were collected since genes may have multiple transcripts[[1](#_ENREF_1)].

**Gene-region expression network (G-R)**

In different regions of the brain, gene expression levels are different, and AHBA provide this fine-grained information. Data from AHBA consisting of six genome-wide gene expression profiles and accompanying anatomical and histological data on the human brain. A three-dimensional neuroanatomic space within each brain can be reconstructed using microarray data combined with anatomical and histological information at each level of partitioning. In total, six gene expression profiles are presented encompassing 3,702 regions in six brains, each containing information for over 58,692 gene probes. By taking advantage of the complementary base pairing principle, gene probes can bind precisely to gene loci and detect their expression levels in genome. To eliminate systematic errors, biological repetitions are required, using at least two probes to measure for each gene. The 58,692 gene probes cover 20,787 unique genes in the human whole genome.

A unified quality control and normalization process is applied to the six gene expression profiles according to an official Allen Institute white paper. This ensures compatibility among the profiles. Based on the processing and normalization of gene probe expression values according to the previous method[[2](#_ENREF_2)], unique gene expression levels in various brain regions were obtained. Here, denoting that $B=\left\{ B_{1}, B_{2}, \ldots, B_{N_{B}} \right\}$ is a set of $N_{B}$ gene probes, $R=\left\{ R_{1}, R_{2}, \ldots, R_{N_{R}} \right\}$ is a set of $N_{R}$ brain regions and $G=\left\{ G_{1}, G_{2}, \ldots, G_{N_{G}} \right\}$ is a set of $N_{G}$ unique genes. The gene probe expression values in each brain region can be represented by a matrix $M_{\mathrm{BR}}\in\mathbb{R}^{N_{B} \times N_{R}}$. The unique genes covered by gene probes can be represented by a matrix $M_{\mathrm{BG}}\in\mathbb{R}^{N_{B} \times N_{G}}$. The expression level of unique genes in each brain region can be represented by a matrix $M_{\mathrm{GR}}\in\mathbb{R}^{N_{G} \times N_{R}}$. Mathematically, these datasets can be defined and calculated as follows:

$$\begin{aligned} M_{\mathrm{BR}}=\left[ \begin{matrix} m_{1, 1} & \cdots& m_{1,N_{R}} \\ \vdots& \ddots& \vdots\\ m_{N_{B}, 1} & \cdots& m_{N_{B}, N_{R}} \end{matrix} \right]\#\left( 1 \right) \end{aligned}$$

$$\begin{aligned} M_{\mathrm{BG}}=\left\{ \begin{aligned} &1 if G_{i} is covered by B_{j} \\ &0 otherwise \end{aligned} \right.\#\left( 2 \right) \end{aligned}$$

$$\begin{aligned} M_{\mathrm{GR}}={M_{\mathrm{BG}}}^{T}\cdot M_{\mathrm{BR}}\#\left( 3 \right) \end{aligned}$$

where $m_{i, j}(i\in N_{G}, j\in N_{R})$ is the expression value of i-th gene probe in j-th brain region. $G_{i}$ is the i-th unique gene and $B_{j}$ is the j-th gene probe.

**Brain parcel-parcel functional connectivity network (P-P)**

Based on the blood oxygen level-dependent (BOLD) signal detected by r-fMRI, neuronal activity can be indirectly collected[[3](#_ENREF_3), [4](#_ENREF_4)]. In different brain regions, BOLD signal presents a spontaneous but coherent low-frequency fluctuations phenomenon. In some studies, this phenomenon has been attributed to the fact that the brain exhibits a modular functional architecture, in which neurons with BOLD signals that are highly similar are clustered together[[5-8](#_ENREF_5)]. The brain functional connectivity (BFC) network is comprised of these modules, also known as parcels in neuroscience. The BFC network used in this study was constructed by r-fMRI data from HCP (dataset version HCP_1200). It includes 1,003 subjects (the person who undergoing r-fMRI scan) and having four complete r-fMRI runs (4,800 total timepoints). Various frameworks are capable of partitioning r-fMRI data into different BFC network for application in different studies. As part of this study, a framework known as Cole-Anticevic Brain-Wide Network Partition (CAB-NP) has been used, which was obtained from the Cole Neurocognition Lab[[7](#_ENREF_7), [8](#_ENREF_8)]. Apart from its excellent robustness, CAB-NP framework combines all brain structures, including both brain cortex and subcortex, into a single large-scale network. In this sense, this framework is more suitable for large-scale gene-disease functional association network studies than others.

Based on the CAB-NP framework, the brain is partitioned into 718 parcels, and the Pearson correlation coefficient is used to determine the potential functional connection between the parcels. Raw data from HCP is loaded and placed into CAB-NP framework using Connectome Workbench tool, which is specifically designed for exploring HCP-generated data[[9](#_ENREF_9)]. Here, denoting that $P=\left\{ P_{1}, P_{2}, \ldots, P_{N_{P}} \right\}$ is a set of $N_{P}$ brain parcels. Mathematically, the brain parcel-parcel functional connectivity network can be represented by a matrix $M_{\mathrm{PP}}\in\mathbb{R}^{N_{P} \times N_{P}}$ as follow:

$$\begin{aligned} M_{\mathrm{PP}}=\left[ \begin{matrix} m_{1, 1} & \cdots& m_{1,N_{P}} \\ \vdots& \ddots& \vdots\\ m_{N_{P}, 1} & \cdots& m_{N_{P}, N_{P}} \end{matrix} \right]\#\left( 4 \right) \end{aligned}$$

where $m_{i, j}(i\in N_{P}, j\in N_{P})$ is the functional connectivity strength of i-th brain parcel with j-th brain parcel.

**Matching relationship between brain parcels and regions (P-R)**

Each part of the brain from the AHBA has its own unique coordinates in the Montreal Neurological Institute (MNI) space, which is the most commonly used spatial coordinate system in neuroimaging[[10](#_ENREF_10)]. In view of the fact that brain parcels may include more than one brain structure, grayordinates were introduced by HCP to unitarily represent these structures[[11](#_ENREF_11)]. Each grayordinate has unique coordinate in the MNI space. As such, brain parcels and regions can be matched by MNI coordinates according to the previous method[[2](#_ENREF_2)]. Mathematically, the matching relationship can be represented by the following matrix:

$$\begin{aligned} M_{\mathrm{PR}}=\left\{ \begin{aligned} &1 if P_{i}\mathrm{and}R_{j} is matched \\ &0 otherwise \end{aligned} \right.\#\left( 5 \right) \end{aligned}$$

where $P_{i,j}(i\in N_{P}, j\in N_{R})$ is the i-th brain parcel and $R_{j}$ is the j-th brain region.

**Brain region-region functional connectivity network (R-R)**

As brain parcels can be matched to brain regions by MNI coordinates, the functional connectivity between brain regions can be converted from the functional connectivity of brain parcels. Mathematically, brain region-region functional connectivity network can be obtained by converting and represented by a matrix $M_{\mathrm{RR}}\in\mathbb{R}^{N_{R} \times N_{R}}$ as follows:

$$\begin{aligned} M_{\mathrm{RR}}={M_{\mathrm{PR}}}^{T}\cdot M_{\mathrm{PP}}\cdot M_{\mathrm{PR}}\#\left( 6 \right) \end{aligned}$$

$$\begin{aligned} M_{\mathrm{RR}}=\left[ \begin{matrix} m_{1, 1} & \cdots& m_{1,N_{R}} \\ \vdots& \ddots& \vdots\\ m_{N_{R}, 1} & \cdots& m_{N_{R}, N_{R}} \end{matrix} \right]\#\left( 7 \right) \end{aligned}$$

where $m_{i, j}(i\in N_{R}, j\in N_{R})$ is the functional connectivity strength of i-th brain region with j-th brain region.

**Gene regulatory networks (G-T)**

Transcriptional factors (TFs) play an important role in regulating gene expression, and each gene has a unique combination of active transcription factors[[12](#_ENREF_12), [13](#_ENREF_13)], which is the reason of gene regulatory network (GRN) is vital to understanding the complex mechanisms controlling gene expression. The GRNdb database provides gene regulatory information by integrating high-quality RNA-seq data taken from various human tissues to construct TFs and target genes networks[[14](#_ENREF_14)]. The datasets in GRNdb were analyzed by SCENIC pipeline[[15](#_ENREF_15)], several studies have demonstrated its comprehensiveness and accuracy in predicting TF and target-genes regulatory relationships[[16-18](#_ENREF_16)].

The GRN used in this study includes 20,274 unique genes and 738 TFs in human genome, with a total of 162,144 TF-target gene regulatory pairs linked by weight scores. The more likely regulatory links are indicated by the higher weight score. Here, denoting that the $T=\left\{ T_{1}, T_{2}, \ldots, T_{N_{T}} \right\}$ is a set of $N_{T}$ TFs, the unique genes are same as in the Allen Human Brain Atlas. Mathematically, GRN can be represented by a matrix $M_{\mathrm{GT}}\in\mathbb{R}^{N_{G} \times N_{T}}$ as follow:

$$\begin{aligned} M_{\mathrm{GT}}=\left[ \begin{matrix} m_{1, 1} & \cdots& m_{1,N_{T}} \\ \vdots& \ddots& \vdots\\ m_{N_{G}, 1} & \cdots& m_{N_{G}, N_{T}} \end{matrix} \right]\#\left( 8 \right) \end{aligned}$$

where $m_{i, j}(i\in N_{G}, j\in N_{T})$ is the weight score of i-th unique gene with j-th TF.

**TF-TF similarities (T-T)**

Homology-based methods can be employed to determine the function of a protein by comparing it to another protein with a known function[[19](#_ENREF_19)]. According to the blast algorithm, the all-versus-all alignment was used to identify the homology between the amino acid sequences of 738 TFs[[20](#_ENREF_20)]. Mathematically, the similarities can be represented by a matrix $M_{\mathrm{TT}}\in\mathbb{R}^{N_{T} \times N_{T}}$ as follow:

$$\begin{aligned} M_{\mathrm{TT}}=\left[ \begin{matrix} m_{1, 1} & \cdots& m_{1,N_{T}} \\ \vdots& \ddots& \vdots\\ m_{N_{T}, 1} & \cdots& m_{N_{T}, N_{T}} \end{matrix} \right]\#\left( 9 \right) \end{aligned}$$

where $m_{i, j}(i\in N_{T}, j\in N_{T})$ is the homology of i-th TF with j-th TF.

**Gene network based on regulatory relationships (G-G)**

In many cases, a TF often binds to different regulatory regions of different genes and usually induces their coordinated expression[[21-23](#_ENREF_21)]. This results in co-expression of genes regulated by the same transcription factor, as well as likely similar biological functions. A gene similarity network based on gene regulatory relationships can be used to reflect functional similarity from the perspective of gene regulation, since gene expression is typically mediated by transcription factors and the GRNdb database provides comprehensive and high-quality gene regulatory relationships. Unlike traditional gene sequence similarity networks[[24](#_ENREF_24), [25](#_ENREF_25)], this gene network consisting up to 20,274 unique genes and integrates regulatory information. Notably, gene regulatory information is derived from RNA-seq data, so the gene network used in this study implicitly includes both the sequence and expression characteristics of genes. Mathematically, the gene network based on regulatory relationships can be calculated and represented by a matrix $M_{\mathrm{GG}}\in\mathbb{R}^{N_{G} \times N_{G}}$ as follows:

$$\begin{aligned} M_{\mathrm{GG}}={M_{\mathrm{GT}}}^{T}\cdot M_{\mathrm{TT}}\cdot M_{\mathrm{GT}}\#\left( 10 \right) \end{aligned}$$

$$\begin{aligned} M_{\mathrm{GG}}=\left[ \begin{matrix} m_{1, 1} & \cdots& m_{1,N_{G}} \\ \vdots& \ddots& \vdots\\ m_{N_{G}, 1} & \cdots& m_{N_{G}, N_{G}} \end{matrix} \right]\#\left( 11 \right) \end{aligned}$$

where $m_{i, j}(i\in N_{G}, j\in N_{G})$ is the functional similarity strength of i-th gene with j-th gene.

**Disease-disease similarity network based on variants (D-D)**

In general, diseases are associated with specific genes, and variations in these genes can result in alterations in gene function[[26-28](#_ENREF_26)]. By computing the number of shared variants between two diseases, a disease-disease similarity network consisting 10,392 diseases was constructed[[29](#_ENREF_29)]. According to the DisGeNET database guidelines, the Jaccard index was used to assess the fraction of shared variants among diseases. Denoting the $D=\left\{ D_{1}, D_{2}, \ldots, D_{N_{D}} \right\}$ is a set of $N_{D}$ diseases and $V=\left\{ V_{1}, V_{2}, \ldots, V_{N_{D}} \right\}$ is a set of variants associated to each disease. For each disease pair, the fraction $J_{D}$ can be calculated as follow:

$$\begin{aligned} J_{D_{i},D_{j}}=\frac{V_{i} \cap V_{j}}{V_{i} \cup V_{j}}\#\left( 12 \right) \end{aligned}$$

where $V_{i}(i\in N_{D})$ and $V_{j}(j\in N_{D})$ are the sets of variants associated to $D_{i}$ and $D_{j}$.

Mathematically, the disease-disease similarity network can be represented by a matrix $M_{\mathrm{DD}}\in\mathbb{R}^{N_{D} \times N_{D}}$ as follow:

$$\begin{aligned} \begin{aligned} M_{\mathrm{DD}}=\left[ \begin{matrix} J_{1, 1} & \cdots& J_{1,N_{D}} \\ \vdots& \ddots& \vdots\\ J_{N_{D}, 1} & \cdots& J_{N_{D}, N_{D}} \end{matrix} \right] \end{aligned}\#\left( 13 \right) \end{aligned}$$

where $J_{i, j}(i\in N_{D}, j\in N_{D})$ is the Jaccard index of i-th disease with j-th disease.

**Gene-disease association network (G-D)**

The DisGeNET database provides comprehensive information on gene-disease associations[[29](#_ENREF_29)]. A large number of scientific experiments have been collected into this database, which is highly useful in network-based research, because it provides accurate and comprehensive gene-disease associations[[2](#_ENREF_2), [30](#_ENREF_30), [31](#_ENREF_31)]. Mathematically, the associations between unique genes and diseases can be represented by a matrix $M_{\mathrm{GD}}\in\mathbb{R}^{N_{G} \times N_{D}}$ as follow:

$$\begin{aligned} M_{\mathrm{GD}}=\left\{ \begin{aligned} &1 if G_{i} associated with D_{j} \\ &0 otherwise \end{aligned} \right.\#\left( 14 \right) \end{aligned}$$

where $G_{i}$ is the i-th unique gene and $D_{j}$ is the j-th disease.

**References**

1. Gonzàlez-Porta M, Frankish A, Rung J et al. Transcriptome analysis of human tissues and cell lines reveals one dominant transcript per gene, Genome Biology 2013;14:R70.

2. Wang W, Han R, Zhang M et al. A network-based method for brain disease gene prediction by integrating brain connectome and molecular network, Briefings in Bioinformatics 2022;23:bbab459.

3. Ogawa S, Lee TM, Kay AR et al. Brain magnetic resonance imaging with contrast dependent on blood oxygenation, Proceedings of the National Academy of Sciences 1990;87:9868-9872.

4. Wang P, Wang J, Michael A et al. White Matter Functional Connectivity in Resting-State fMRI: Robustness, Reliability, and Relationships to Gray Matter, Cerebral Cortex 2021;32:1547-1559.

5. Bullmore E, Sporns O. Complex brain networks: graph theoretical analysis of structural and functional systems, Nature Reviews Neuroscience 2009;10:186-198.

6. Ji JL, Spronk M, Kulkarni K et al. Mapping the human brain's cortical-subcortical functional network organization, NeuroImage 2019;185:35-57.

7. Power Jonathan D, Cohen Alexander L, Nelson Steven M et al. Functional Network Organization of the Human Brain, Neuron 2011;72:665-678.

8. Yeo BTT, Krienen FM, Sepulcre J et al. The organization of the human cerebral cortex estimated by intrinsic functional connectivity, Journal of Neurophysiology 2011;106:1125-1165.

9. Marcus D, Harwell J, Olsen T et al. Informatics and Data Mining Tools and Strategies for the Human Connectome Project, Frontiers in neuroinformatics 2011;5.

10. Laird AR, Robinson JL, McMillan KM et al. Comparison of the disparity between Talairach and MNI coordinates in functional neuroimaging data: Validation of the Lancaster transform, NeuroImage 2010;51:677-683.

11. Glasser MF, Smith SM, Marcus DS et al. The Human Connectome Project's neuroimaging approach, Nature Neuroscience 2016;19:1175-1187.

12. Karlebach G, Shamir R. Modelling and analysis of gene regulatory networks, Nature Reviews Molecular Cell Biology 2008;9:770-780.

13. Latchman DS. Transcription factors: An overview, The International Journal of Biochemistry & Cell Biology 1997;29:1305-1312.

14. Fang L, Li Y, Ma L et al. GRNdb: decoding the gene regulatory networks in diverse human and mouse conditions, Nucleic Acids Research 2020;49:D97-D103.

15. Aibar S, González-Blas CB, Moerman T et al. SCENIC: single-cell regulatory network inference and clustering, Nature Methods 2017;14:1083-1086.

16. Cao J, O’Day DR, Pliner HA et al. A human cell atlas of fetal gene expression, Science 2020;370:eaba7721.

17. Ramachandran P, Dobie R, Wilson-Kanamori JR et al. Resolving the fibrotic niche of human liver cirrhosis at single-cell level, Nature 2019;575:512-518.

18. Rambow F, Rogiers A, Marin-Bejar O et al. Toward Minimal Residual Disease-Directed Therapy in Melanoma, Cell 2018;174:843-855.e819.

19. Sinha S, Eisenhaber B, Lynn AM. Predicting Protein Function Using Homology-Based Methods. In: Shanker A. (ed) Bioinformatics: Sequences, Structures, Phylogeny. Singapore: Springer Singapore, 2018, 289-305.

20. Altschul SF, Gish W, Miller W et al. Basic local alignment search tool, Journal of Molecular Biology 1990;215:403-410.

21. Davidson EH. Genomic regulatory systems: in development and evolution. Elsevier, 2001.

22. Latchman D. Gene regulation. Taylor & Francis, 2007.

23. Marco A, Konikoff C, Karr TL et al. Relationship between gene co-expression and sharing of transcription factor binding sites in Drosophila melanogaster, Bioinformatics 2009;25:2473-2477.

24. Li G, Zhang P, Sun W et al. Bridging-BPs: a novel approach to predict potential drug–target interactions based on a bridging heterogeneous graph and BPs2vec, Briefings in Bioinformatics 2022;23.

25. Guo Z-H, You Z-H, Huang D-S et al. A learning based framework for diverse biomolecule relationship prediction in molecular association network, Communications Biology 2020;3:118.

26. Fishel R, Lescoe MK, Rao MRS et al. The human mutator gene homolog MSH2 and its association with hereditary nonpolyposis colon cancer, Cell 1993;75:1027-1038.

27. Frazer J, Notin P, Dias M et al. Disease variant prediction with deep generative models of evolutionary data, Nature 2021;599:91-95.

28. Reva B, Antipin Y, Sander C. Predicting the functional impact of protein mutations: application to cancer genomics, Nucleic Acids Research 2011;39:e118-e118.

29. Piñero J, Saüch J, Sanz F et al. The DisGeNET cytoscape app: Exploring and visualizing disease genomics data, Computational and Structural Biotechnology Journal 2021;19:2960-2967.

30. Rao A, Vg S, Joseph T et al. Phenotype-driven gene prioritization for rare diseases using graph convolution on heterogeneous networks, BMC Medical Genomics 2018;11:57.

31. Yang K, Lu K, Wu Y et al. A network-based machine-learning framework to identify both functional modules and disease genes, Human Genetics 2021;140:897-913.
